# Canonical NF-κB signaling in myeloid cells promotes lung metastasis in a mouse breast cancer model

**DOI:** 10.18632/oncotarget.24697

**Published:** 2018-03-30

**Authors:** Tobias Neumann, Özge Canli, Florian R. Greten

**Affiliations:** ^1^ Institute for Tumor Biology and Experimental Therapy, Georg-Speyer-Haus, 60596 Frankfurt, Germany; ^2^ German Cancer Consortium (DKTK) and German Cancer Research Center (DKFZ), 69120 Heidelberg, Germany

**Keywords:** breast cancer, lung metastasis, inflammation, NF-κB, myeloid cells

## Abstract

An inflammatory tumor microenvironment is a common characteristic of solid tumors. It is the result of a complex interplay between tumor cells, tumor infiltrating immune cells and other stromal cells. Myeloid cells in the tumor microenvironment are considered major drivers of tumor progression and metastasis and increased numbers of these cells are associated with poor prognosis in various cancer patients. The transcription factor NF-κB is considered the master regulator of inflammatory gene expression and immune cell function. Its activation in various cells of the tumor microenvironment contributes essentially to tumorigenesis. In the present study, the role of canonical NF-κB signaling in myeloid cells in metastatic breast cancer was addressed by myeloid-specific deletion of *Ikkβ* in the MMTV polyoma middle T (PyMT) mouse model. *Ikkβ* deletion in myeloid cells did not affect primary mammary tumor growth but significantly reduced lung metastasis. While dissemination from the primary tumor was unaltered, myeloid-specific *Ikkβ* loss resulted in a strong up-regulation of pro-inflammatory genes and changes in immune cell populations in the lung, creating a tumor-suppressive microenvironment at the distant site. Thus, canonical NF-κB signaling in myeloid cells creates a permissive lung microenvironment that supports breast to lung metastasis.

## INTRODUCTION

Metastatic disease is the leading cause of death in patients with breast cancer and other malignancies. The high mortality reflects the limited treatment options available for progressed mammary carcinoma and highlights the need for new therapeutic approaches. An inflammatory microenvironment is an integral part of basically all tumors, even when they are not initiated by chronic inflammation. The reciprocal interactions between the inflammatory microenvironment and the tumor cells have a profound effect on tumor growth, metastasis and treatment resistance [1, 2]. Despite the progress that has been made in recent years, our understanding of how the microenvironment affects tumor progression and metastatic disease is incomplete and still requires further investigation.

Tumor cells have to overcome several obstacles and undergo a multi-step process called the metastatic cascade before giving rise to metastasis. First, they have to survive and thrive in the primary tumor and secrete factors that induce the pre-metastatic niche. Subsequently, they have to locally invade and enter the circulation to escape the primary tumor. Upon arrival at the metastatic site, the disseminated tumor cells have to egress from the blood stream, seed the tissue and expand to form metastases [3].

All steps of the metastatic cascade are strongly influenced by the immune system and the local microenvironment [1]. On the one hand, tumor cells have to constantly evade growth suppression and elimination by the anti-tumor immune response, especially cytotoxic T-cells and NK cells [4–7]. On the other hand, immune cells, in particular myeloid cells, are recruited and educated by tumor cells to actively facilitate their progression along the metastatic cascade. Macrophages comprise the most abundant myeloid cell type in human breast tumors [8] and their abundance in primary tumors is associated with poor prognosis [9, 10]. Pre-clinical studies have provided insights into the numerous ways macrophages can promote tumor progression and metastasis. They enhance tumor cell survival and proliferation by paracrine factors [11–13], trigger the angiogenic switch [14–17], promote local invasion [18, 19] and suppress CD8^+^ T-cells [9, 20]. Moreover, macrophages support tumor cell intravasation at the primary tumor [21] as well as extravasation at the distant site [22] and they have been shown to provide survival signals to tumor cells which colonize the distant site [23].

In parallel, intratumoral myeloid Ly6G^+^ cells drive tumor progression by stimulating tumor cell proliferation [24] and tumor neo-vascularization [25, 26], as well as inhibiting T-cell responses [27–29]. Furthermore, Ly6G^+^ cells are recruited to the metastatic site prior to colonization and establish the pre-metastatic niche, a microenvironment that is favorable for metastasis-initiating cells [30–33].

In human breast cancer specimens, NF-κB activation has been reported in both tumor cells as well as in the tumor stroma [34]. In addition, several cytokines which are known activators of NF-κB, including IL-1β, TNFα and IL-6, were reported to be upregulated in mammary tumors [35]. Moreover, previous studies of our lab using *in vivo* models of carcinogen-induced colon cancer [11, 12] demonstrated a tumor-promoting role of NF-κB signaling in myeloid cells during tumor promotion and progression. Given these reports and the high abundance of myeloid cells in mammary tumors [8, 9], we hypothesized that NF-κB signaling in myeloid cells might drive tumor progression in breast cancer. To test our hypothesis, we specifically deleted *Ikkβ* in myeloid cells in a well-established mouse model of metastatic breast cancer. The IKKβ subunit of the IKK complex is required for canonical NF-κB. Its activation leads to IκBα phosphorylation which upon ubiquitination is degraded by the proteasome. Subsequently, this triggers the release of NF-κB dimers that can now translocate to the nucleus to bind DNA and to induce transcription [36].

We show that IKKβ dependent NF-κB activation in myeloid cells is dispensable for primary tumor growth but required for establishing a lung microenvironment that supports the development of metastases.

## RESULTS

To study the role of canonical NF-κB signaling in myeloid cells in breast cancer we crossed LysM-Cre/*Ikkβ^F/F^* (*Ikkβ^Δmye^)* mice [11] with mice that carry the polyoma middle T oncogene under the control of the MMTV promoter (MMTV PyMT) [37]. *Ikkβ^Δmye^* mice have a deletion of *Ikkβ* in myeloid cells preventing canonical NF-κB activation [11], whereas MMTV-PyMT mice develop spontaneous mammary carcinomas that metastasize with high incidence to the lung [37].

**Figure 1:**
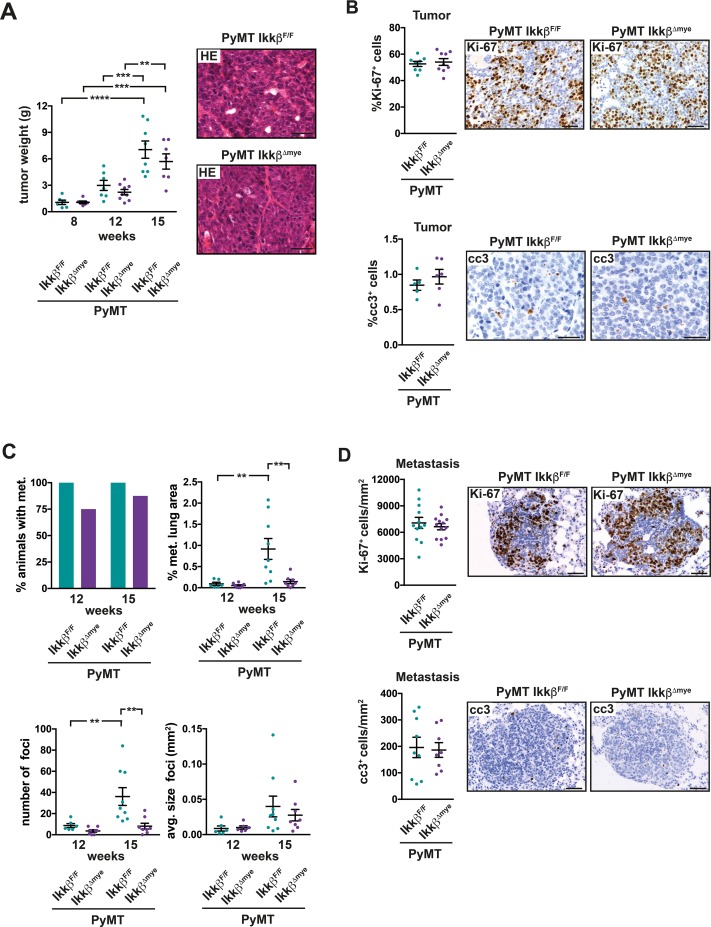
Deletion of *Ikkβ* in myeloid cells does not affect primary tumor growth but suppresses lung metastasis in the PyMT breast cancer model **(A)** Combined weight of all mammary tumors per animal from PyMT *Ikkβ^F/F^* and PyMT *Ikkβ^Δmye^* mice at 8, 12 and 15 weeks of age (each n≥6) and representative H&E-stained primary tumor tissue at 15 weeks of age. **(B)** Percentage of Ki-67 positive (Ki-67^+^) cells and cleaved caspase 3 positive (cc3^+^) in tumors of PyMT *Ikkβ^F/F^* and PyMT *Ikkβ^Δmye^* mice at 15 weeks of age. Two tumors per animal were analyzed, depicted is the mean for each animal. Ki-67^+^ cells were quantified in a full section of the tumor (n≥8); cc3^+^ cells were quantified in 6 random 20x fields (n≥5). **(C)** Percentage of animals with lung metastasis, percentage of metastatic area, number and average size of metastatic foci in the lungs of PyMT *Ikkβ^F/F^* and PyMT *Ikkβ^Δmye^* mice at 12 and 15 weeks of age (each n≥6). **(D)** Ki-67^+^ cells and cc3^+^ cells per mm^2^ metastasis in metastatic foci from n≥5 PyMT *Ikkβ^F/F^* and PyMT *Ikkβ^Δmye^* mice at 15 weeks of age. Data are mean ± SEM. ^**^p≤0,01 ^***^p≤0,001 ^****^p.≤0,0001. Scale bar is 0,05mm.

In the resulting PyMT *Ikkβ^Δmye^* mice primary tumor burden was not significantly altered compared to *Ikkβ*-proficient PyMT littermate controls and histological appearance of tumors from both genotypes was similar (Figure 1A). Consistently, proliferation and apoptosis of tumor cells determined by Ki-67 and cleaved caspase 3 immunohistochemistry, respectively, were comparable in both genotypes in the primary tumor at 15 weeks of age (Figure 1B). However, while all PyMT *Ikkβ^F/F^* animals had developed microscopically visible metastases at 12 weeks of age in the lung, 25% of PyMT *Ikkβ^Δmye^* mice were metastasis free (Figure 1C). At 15 weeks of age, the number of lung metastases in PyMT *Ikkβ^F/F^* control animals was more than four times higher compared to PyMT *Ikkβ^Δmye^* mice (Figure 1C). The size (Figure 1C) of established metastatic foci, nevertheless, was similar in PyMT *Ikkβ^F/F^* and PyMT *Ikkβ^Δmye^* animals, as was the number of Ki-67 and cleaved caspase 3 positive metastatic cells (Figure 1D). Thus, deletion of *Ikkβ* in myeloid cells does not affect primary tumor growth but potently suppresses formation of metastatic foci in the lung.

**Figure 2:**
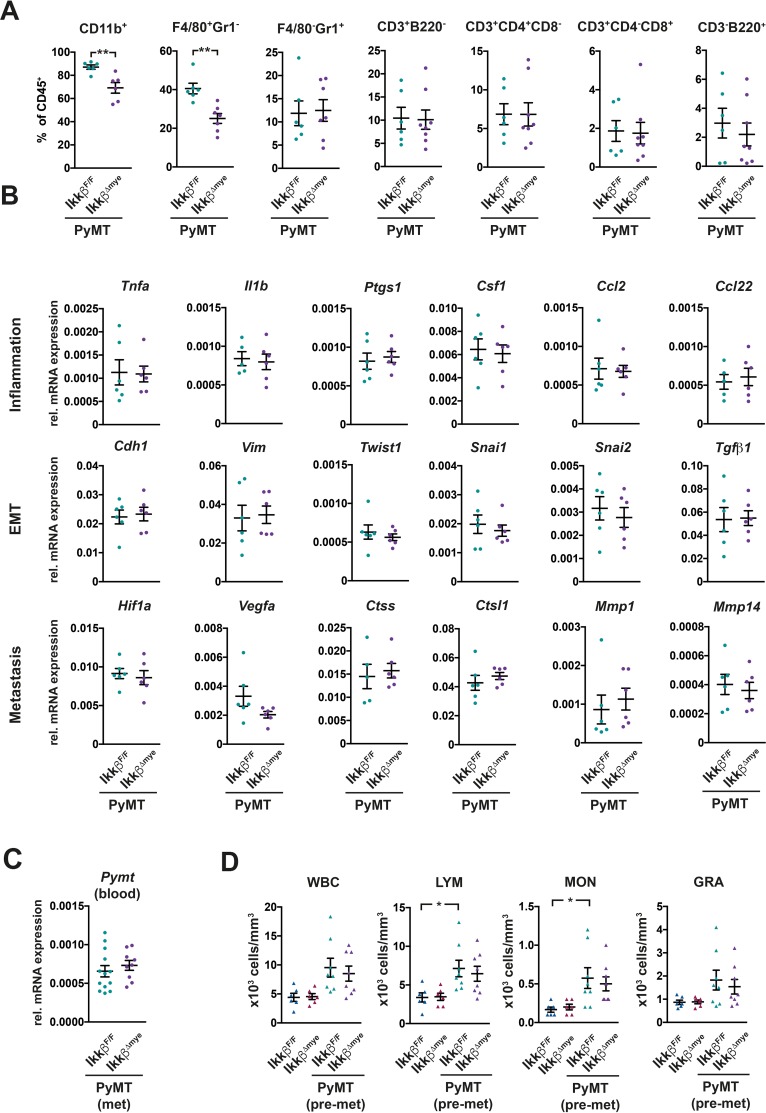
Deletion of *Ikkβ* in myeloid cells has only minor effects on the primary tumor microenvironment and does not affect dissemination **(A)** Flow cytometric analysis of immune cell populations in tumors of PyMT *Ikkβ^F/F^* and PyMT *Ikkβ^Δmye^* mice at 12 weeks of age. Two or more tumors per animal were analyzed, depicted is the mean for each animal (n≥6). **(B)** Quantitative PCR of the indicated genes from tumor tissue of PyMT *Ikkβ^F/F^* and PyMT *Ikkβ^Δmye^* mice at 12 weeks of age. Two tumors per animal were analyzed, depicted is the mean for each animal (n≥5). **(C)** *Pymt* oncogene expression in the blood of PyMT *Ikkβ^F/F^* and PyMT *Ikkβ^Δmye^* mice (n≥9) at 15 weeks of age. **(D)** Immune cells in the peripheral blood of tumor free *Ikkβ^F/F^* and *Ikkβ^Δmye^* mice as well as tumor-bearing PyMT *Ikkβ^F/F^* and PyMT *Ikkβ^Δmye^* mice (n≥6) at 8 weeks of age. Depicted are total white blood cells (WBC), lymphocytes (LYM), monocytes (MON) and granulocytes (GRA). Data are mean ± SEM. ^**^p≤0,01

Immune cells shape the local microenvironment during tumorigenesis and are important modulators of the metastatic cascade [1, 38]. To determine whether deletion of *Ikkβ* in myeloid cells affects the microenvironment in the primary tumor, we characterized tumor infiltrating immune cell populations by flow cytometry (Figure 2A, Supplementary Figure 1). Additionally, we determined the expression of several genes related to inflammation, epithelial to mesenchymal transition (EMT) and metastasis by RT-qPCR (Figure 2B). Myeloid cells, specifically CD11b^+^ F4/80^+^ Gr1^−^ tumor-associated macrophages (TAMs) were by far the most abundant immune cell population in tumors of both PyMT *Ikkβ^Δmye^* and PyMT *Ikkβ^F/F^* control animals (Figure 2A). Yet, in tumors of PyMT *Ikkβ^Δmye^* animals, the proportion of viable F4/80^+^ Gr1^−^ macrophages was significantly reduced. The proportion of F4/80^−^ Gr1^+^ granulocytes, CD3^+^ T-cells and B220^+^ B-cells, on the other hand, was comparable to PyMT *Ikkβ^F/F^* controls (Figure 2A). Interestingly, we could not detect any differences in mRNA expression of genes involved in inflammation and metastasis in tumors from PyMT *Ikkβ^Δmye^* and PyMT *Ikkβ^F/F^* controls (Figure 2B).

To determine whether dissemination of tumor cells from the primary site was altered in PyMT *Ikkβ^Δmye^* mice, we quantified circulating tumor cells by measuring the mRNA expression of the *Pymt* oncogene in the blood. We found no difference in *Pymt* expression in the blood between PyMT *Ikkβ^Δmye^* and PyMT *Ikkβ^F/F^* control mice (Figure 2C). Mobilization of immune cells in the peripheral blood in response to the primary tumor was unaffected by myeloid-specific loss of *Ikkβ* as well (Figure 2D).

Collectively, these findings suggest that loss of *Ikkβ* in myeloid cells has only minor influence on primary tumor growth and the primary tumor microenvironment and is dispensable for the early events of the metastatic cascade. Thus, we hypothesized that metastasis in PyMT *Ikkβ^Δmye^* might be impaired at the end of the metastatic cascade at the distant site. Therefore, we tried to identify alterations in the lungs of PyMT *Ikkβ^Δmye^* mice that might account for the reduction in metastatic foci.

**Figure 3:**
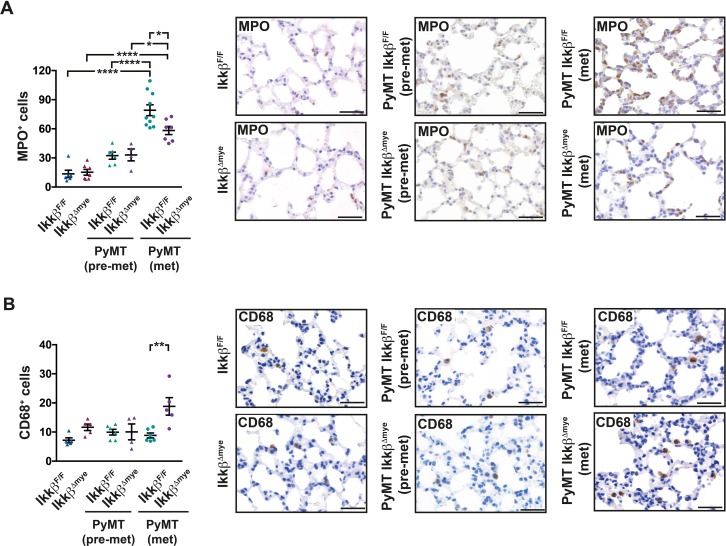
Lungs of PyMT *Ikkβ^Δmye^* mice have altered immune cell infiltration Number of **(A)** MPO^+^ cells or **(B)** CD68^+^ cells per field in lungs of *Ikkβ^F/F^* and *Ikkβ^Δmye^* mice (tumor free; n=5) or PyMT *Ikkβ^F/F^* and PyMT *Ikkβ^Δmye^* mice at 8 weeks (pre-metastatic; n≥4) or 15 weeks of age (metastatic; n≥5), respectively. 9 random 40x fields per animal were analyzed. Data are mean ± SEM. ^*^p≤0,05; ^**^p≤0,01; ^****^p≤0,0001. Scale bar is 0,05mm.

Since myeloid cells at the distant site have been suggested as crucial modulators of metastasis [6, 22, 30, 32, 39], we determined the numbers of granulocytes and macrophages in the lung parenchyma. Immunohistochemical analysis of MPO^+^ cells revealed a strong and gradual increase of granulocytes in the lung parenchyma of tumor bearing mice compared to tumor free animals in both genotypes (Figure 3A). However, lungs from PyMT *Ikkβ^Δmye^*mice in the metastatic stage displayed significantly reduced numbers of MPO^+^ granulocytes compared to PyMT *Ikkβ^F/F^* controls (Figure 3A). In contrast, the number of CD68^+^ macrophages in the lung parenchyma of metastatic animals was significantly increased upon myeloid *Ikkβ* deletion (Figure 3B). Interestingly, although containing lower numbers of CD68^+^ cells compared to metastatic PyMT *Ikkβ^Δmye^* mice, lungs from tumor free *Ikkβ^Δmye^* animals showed a clear trend towards increased presence of CD68^+^ cells as well.

**Figure 4:**
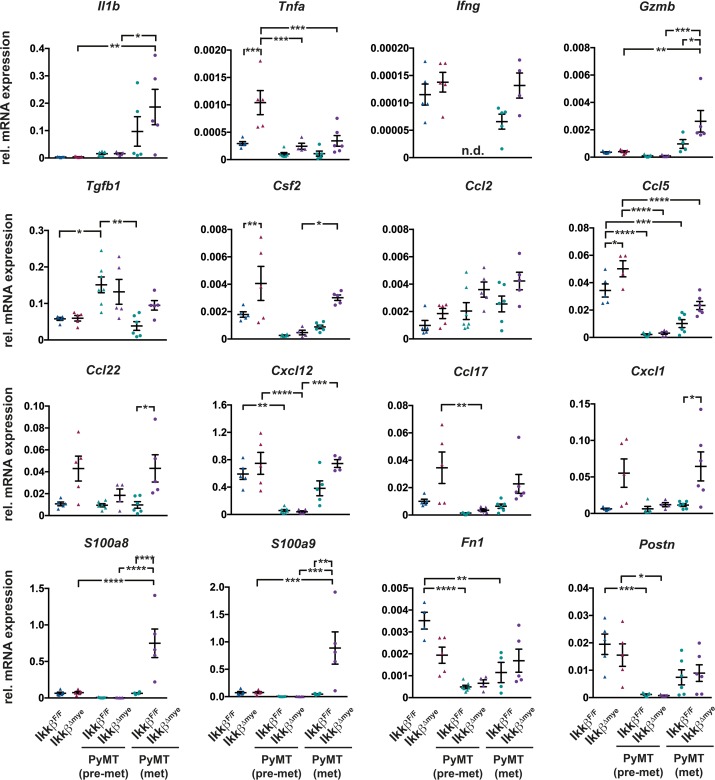
Deletion of *Ikkβ* in myeloid cells causes a pro-inflammatory lung microenvironment Quantitative PCR of the indicated genes from lung tissue of *Ikkβ^F/F^* and *Ikkβ^Δmye^* mice (tumor free; n=5) or PyMT *Ikkβ^F/F^* and PyMT *Ikkβ^Δmye^* mice at 8 weeks (pre-metastatic; n≥4) or 15 weeks of age (metastatic; ≥4), respectively. Data are mean ± SEM ^*^p≤0,05; ^**^p≤0,01; ^***^p≤0,001; ^****^p≤0,0001

To further characterize the alterations in the lung microenvironment upon myeloid-specific loss of *Ikkβ*, we analyzed the expression of genes related to inflammation and the pre-metastatic niche in lungs from tumor free mice, pre-metastatic mice (8 weeks) and metastatic mice (15 weeks). Deletion of *Ikkβ* in myeloid cells resulted in marked changes in expression of genes encoding numerous cytokines and chemokines in the lung (Figure 4A). Some of those genes were differentially expressed only in animals at the metastatic stage while others were also altered in tumor free animals. The subset of genes upregulated in lungs of both tumor free and metastatic *Ikkβ*-deficient animals comprised *Tnfa*, *Ccl5*, *Ccl17*, *Ccl22*, *Cxcl1* and *Csf2*, although not all changes reached significance. However, only metastatic PyMT *Ikkβ^Δmye^* animals showed significant upregulation of *GrzmB*, *S100a8*, *S100a9* and a clear trend towards increased expression of *Tgfb1* and *Ifng* compared to PyMT *Ikkβ^F/F^* controls. Interestingly, expression of most genes was attenuated at 8 weeks of age in pre-metastatic lungs in both *Ikkβ*-proficient and deficient mice and only *Tgfb1* was higher expressed in both genotypes compared to tumor free and metastatic animals.

Some of the inflammatory mediators that were found elevated in the lungs of tumor-free *Ikkβ^Δmye^* animals (Figure 4A) also showed a trend towards slightly increased expression in the mammary glands of tumor-free *Ikkβ^Δmye^* animals and *Il1b* expression was significantly higher (Supplementary Figure 2).

**Figure 5:**
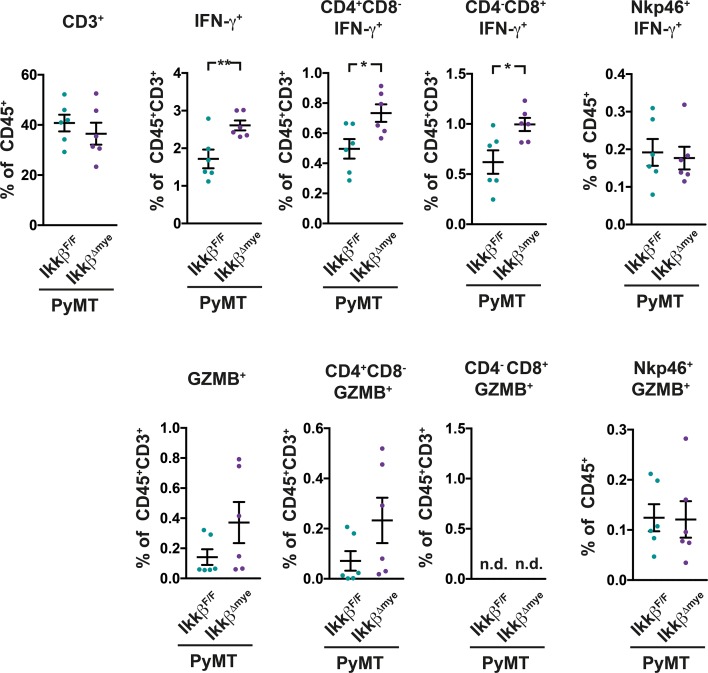
PyMT *Ikkβ^Δmye^* mice have increased levels of activated T-cells in their lungs IFN-γ and Granzyme B expression in mononuclear cells from lungs of PyMT *Ikkβ^F/F^* and PyMT *Ikkβ^Δmye^* mice was determined at 11 weeks of age (n=6) by FACS. Data are mean ± SEM ^*^p≤0,05; ^**^p≤0,01

The elevated mRNA expression of *Ifng* and *GrzmB* in metastatic lungs of PyMT *Ikkβ^Δmye^* animals suggested an increased presence of cytotoxic lymphocytes in that might confer increased protection from metastasis. To determine whether this is was the case, mononuclear cells from the lung were analyzed for IFN-γ and Granzyme B expression by FACS (Figure 5, Supplementary Figure 3). In lungs of PyMT *Ikkβ^Δmye^* mice we found significant more IFN-γ producing CD4^+^ and CD8^+^ T-cells compared to PyMT *Ikkβ^F/F^* controls (Figure 5). Moreover, PyMT *Ikkβ^Δmye^* mice showed a trend towards increased presence of Granzyme B expressing CD4^+^ T-cells in their lungs. Granzyme B expressing CD8^+^ cells on the other hand were only very rarely observed in mice of either genotype. The numbers of IFN-γ and Granzyme B expressing Nkp46^+^ Nk-cells were comparable in PyMT *Ikkβ^F/F^* and PyMT *Ikkβ^Δmye^* mice (Figure 5).

**Figure 6:**
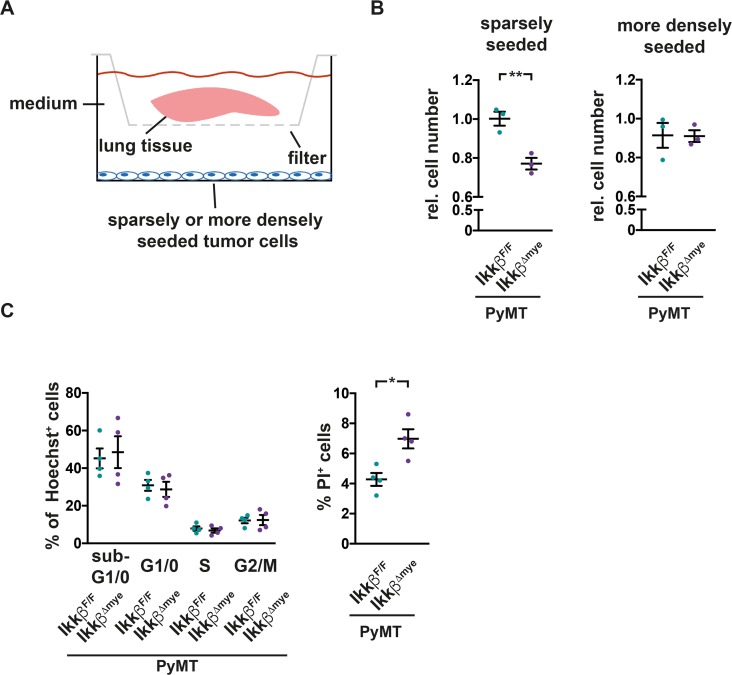
Soluble factors in lungs of PyMT *Ikkβ^Δmye^* mice suppress tumor cell survival **(A)** TS1 tumor cells, seeded sparsely (15.000 cells/well) or more densely (50.000 cells/well) were co-cultured in serum-reduced medium with lung tissue from 12 weeks old, early metastatic PyMT *Ikkβ^F/F^* or PyMT *Ikkβ^Δmye^* mice in a transwell system for 16h (pore diameter 0,4μM). Lung tissue was removed and cell numbers of co-cultured cells were analyzed 24h later. **(B)** Depicted are relative cell numbers of TS1 cells after co-culture from three independent experiments. Each condition was measured with four to five replicates per experiment. **(C)** Cell cycle stage distribution and viability of sparsely seeded TS1 cells after co-culture were determined by Hoechst 33342 and Propidium iodide (PI) staining in FACS. Depicted are two independent experiments with two replicates each. Data are mean ± SEM ^*^p≤0,05; ^**^p≤0,01.

To test whether the pro-inflammatory lung microenvironment in PyMT *Ikkβ^Δmye^* mice has indeed tumor suppressive properties, we co-cultured PyMT tumor-derived TS1 cells in serum-reduced medium with lung tissue from either PyMT *Ikkβ^Δmye^* mice or PyMT *Ikkβ^F/F^* controls in a transwell system. The transwell system allows exchange of soluble factors but prevents physical contact (Figure 6A). When seeded at low density to mimic micrometastatic conditions, TS1 tumor cells co-cultured with lung tissue from PyMT *Ikkβ^Δmye^* mice showed a significant 25% reduction in growth compared to cells co-cultured with lung tissue from PyMT *Ikkβ^F/F^* mice (Figure 6B). Yet, when seeded at a higher density but still below confluence, no suppressive effect on the tumor cells was observed (Figure 6B). Sparse TS1 cells after co-culture were further characterized by FACS in terms of cell cycle progression and viability by staining with Hoechst 33342 and propidium iodide (PI). TS1 cells showed comparable cell cycle distribution after co-culture with lung tissue from PyMT *Ikkβ^F/F^* or PyMT *Ikkβ^Δmye^* mice. However, TS1 cells co-cultured with lung tissue from PyMT *Ikkβ^Δmye^* mice showed a significant increase in the fraction of PI^+^ dead cells (Figure 6C). Thus, loss of *Ikkβ* in myeloid cells leads to a tumor suppressive pro-inflammatory microenvironment in the lung.

## DISCUSSION

Our data reveals canonical NF-κB signaling in myeloid cells as an important mediator of breast to lung metastasis by regulating the microenvironment at the distant site. While deletion of *Ikkβ* in myeloid cells in the PyMT breast cancer model does not have a major impact on primary tumor growth and the primary tumor microenvironment it significantly reduces lung metastasis. Expression of metastasis-related genes in the primary tumor and comparable numbers of circulating tumor cells in PyMT *Ikkβ^F/F^* and PyMT *Ikkβ^Δmye^* indicate that the observed reduction in lung metastasis is not a consequence of altered dissemination from the primary tumor. Concomitantly, lungs of PyMT *Ikkβ^Δmye^* animals exhibit a pro-inflammatory signature that comprises numerous chemokines and cytokines that most likely shape a tumor suppressive environment. While the individual role of these inflammatory mediators is not clear, their integrated effects are tumor-suppressive, as demonstrated in co-culture assays. Some of the factors upregulated in lungs of PyMT *Ikkβ^Δmye^* mice have known cytotoxic properties. For instance, IFN-γ and Granzyme B have been described as potent effector molecules of the anti-cancer defense that can inhibit metastasis [5, 40–42]. TGFβ1 and TNFα are pleiotropic cytokines, which can have both tumor promoting and tumor suppressive effects depending on the context [43–45]. It is possible that in the lungs of PyMT *Ikkβ^Δmye^* mice they directly or indirectly contribute to a tumor hostile environment.

Strikingly, we observed a reduced number of metastatic foci but no difference in their size, proliferation or apoptosis. Thus, once a metastatic focus is successfully established and has progressed to a certain size, it is unaffected by the altered lung microenvironment in PyMT *Ikkβ^Δmye^* mice. This might reflect particular vulnerability of single metastasis-initiating cells compared to tumor cells in the bulk of an established metastasis. Our findings in an *in vitro* co-culture system, where factors secreted from lungs of pre-metastatic PyMT *Ikkβ^Δmye^* mice have suppressive properties only on sparse tumor cells but not on more confluent cells support this notion.

*Ikkβ*-deficiency in myeloid cells leads to the upregulation of pro-inflammatory markers in the lung of tumor free and metastatic mice. Yet, while partly overlapping, there are marked differences in the pro-inflammatory signature in the lungs of tumor free *Ikkβ^Δmye^* and metastatic PyMT *Ikkβ^Δmye^* mice. These differences might be the result of primary tumor-induced systemic effects on the lung microenvironment during the later stages of tumorigenesis. Alternatively, but not mutually exclusive, the particular microenvironment in metastatic PyMT *Ikkβ^Δmye^* mice might develop due to activation of immune cells in the lung when encountering metastasized tumor cells.

The changes in cytokine expression in lungs of PyMT *Ikkβ^Δmye^* mice are accompanied by alterations in immune cell populations. We observed a significant increase in IFN-γ producing CD4^+^ and CD8^+^ T-cells in the lung of PyMT mice *Ikkβ^Δmye^*, which may be responsible for the death of metastasis initiating cells. Also, the number of CD68^+^ macrophages was elevated in the lungs of PyMT *Ikkβ^Δmye^* mice. These cells might reflect bone marrow-derived macrophages (BMDMs) recruited in response to chemotactic cues [22]. A certain macrophage subset has been reported to promote extravasation of tumor cells during metastatic seeding [22, 46]. Yet, it has become clear in recent years that the functional polarization of immune cell populations determine their tumor-promoting or suppressive properties [47, 48]. Intriguingly, targeting NF-κB signaling in macrophages can polarize them towards a more cytotoxic phenotype [49].

Before onset of metastasis, tumor cells in the primary tumor secrete systemically acting soluble factors that prime distant tissues for metastasis. The metastatic niche provides a microenvironment that facilitates seeding and outgrowth of metastasis-initiating cells. Earlier studies have emphasized the importance of granulocytes for making the distant site more susceptible to metastatic seeding [30, 31, 33, 39]. Granulocytes have been reported to promote outgrowth of metastasis-initiating cells through leukotriene secretion [30], enhanced retention of circulating tumor cells by neutrophil extracellular traps [31, 50]. Moreover, granulocytic myeloid cells can suppress anti-tumor immunity and thereby contribute to metastasis [39, 51–53]. In line with these reports, we observed gradual recruitment of granulocytic cells to the lung with progressing tumorigenesis. Interestingly, this recruitment was significantly impaired in PyMT *Ikkβ^Δmye^* mice at the metastatic stage. Thus, impaired granulocyte recruitment to lungs of PyMT *Ikkβ^Δmye^* mice might contribute to reduced lung metastasis by affecting the metastatic niche and T-cell activation in the lung.

Two previous studies analyzed the role of NF-κB signaling in myeloid cells in experimental lung colonization assays. Myeloid-specific deletion of *Ikkβ* strongly increased experimental lung colonization using Braf^V600E^/Pten^−/−^ or B16F0 melanoma cells presumably due to impaired anti-tumor function of macrophages [54]. Moreover, enforced NF-κB activation in myeloid cells through inducible overexpression of *Ikkβ* under the control of the *Csf1r* promoter inhibited experimental colonization of lungs with breast cancer cells [55]. However, in contrast to tumor cell injection into the tail vein the spontaneous PyMT model should be considered as a more adequate model of lung colonization as it also considers tumor evolution. Unlike tail vein injection, it takes into account potential systemic effects that a spontaneously developing primary tumor might have on metastasizing tumor cells and especially at the pre-metastatic niche. For instance, myeloid cells, which are normally recruited to the lung before onset of metastasis are not present in tail vein injection experiments and their potential effects are therefore neglected. The influence of other metastasis-regulating factors of the pre-metastatic niche is likewise lacking. Furthermore, different myeloid cell populations are affected when driving Cre recombinase expression using the *Csf1r* promoter compared to the *Lysozyme M* promoter. Unlike the *Lysozyme M* promoter, the *Csf1r* promoter is not active in mature granulocytes [11, 56–58].

In a colitis-associated colon cancer model, we previously found that deletion of *Ikkβ* in myeloid cells reduces the incidence as well as the size of adenomas due to decreased expression of pro-inflammatory cytokines which promote tumor cell proliferation [11]. On the other hand, in a sporadic colon carcinoma model, deletion of *Ikkβ* in myeloid cells reduced prevented lymph node metastasis but did not affect primary tumor growth [12]. In PyMT *Ikkβ^Δmye^* mice, primary tumor burden was unaltered but the formation of metastatic foci at the distant site was impaired. In contrast, in melanoma and lung cancer models NF-κB signaling in myeloid cells seems to confer a tumor-suppressive role [54]. Thus, myeloid-specific loss of *Ikkβ* can have diverse effects on tumorigenesis, underscoring the complex, context-dependent role of NF-κB signaling in different cancer models and during different stages of tumorigenesis.

Therapeutic targeting of immune cells and other stromal cells in the tumor microenvironment might be advantageous over interfering with tumor cell intrinsic mechanism since they are genetically more stable and therefore less likely to develop resistance [59, 60]. Due to the pivotal role of NF-κB in the inflammatory tumor microenvironment, IKKβ as a central component of the signaling pathway is in principle a promising drug target for cancer therapy. However, due to the multifaceted role of IKKβ in myeloid cells in tumorigenesis, a clear definition is required in which context patients can profit from targeting NF-κB. Our findings suggest that it might be beneficial to target canonical NF-κB in myeloid cells in metastatic breast cancer.

## MATERIALS AND METHODS

### Ethics statement

The investigation has been conducted in accordance to national and international guidelines and has been approved by the authors' institutional review board.

### Animals

MMTV PyMT [37] (The Jackson Laboratory) and LysM-Cre/*Ikkβ^F/F^* (*Ikkβ^Δmye^*) mice [11] have been described previously. The two strains, both on FvB background, were crossed to generate PyMT *Ikkβ^Δmye^* mice. Genotyping of mice was performed with the following primers:

**Table:** 

Cre recombinase fwd	ACCTGAAGATGTTCGCGATTATCT
Cre recombinase rev	ACCGTCAGTACGTGAGATATCTT
IkkβΔ (“lox”) fwd	CACAGTGCCCACATTATTTAGATA
IkkβΔ (“lox”) rev	GTCTTCAACCTCCCAAGCCTT
PyMT (control) fwd	CAAATGTTGCTTGTCTGGTG
PyMT (control) rev	GTCAGTCGAGTGCACAGTTT
PyMT (transgene) fwd	GGAAAGTCACTAGGAGCAGGG
PyMT (transgene) rev	GGAAGCAAGTACTTCACAAGGG

### Determining primary tumor burden

To determine tumor weight, mice were sacrificed and the complete mammary tumor tissue was isolated and weighted.

### Histological analysis

For histological analysis, tissue was fixed over night at 4°C in 4% paraformaldehyde in PBS. After fixation, tissue was dehydrated, embedded in paraffin and sectioned. Tissue sections were deparaffinized, rehydrated and stained with Hämatoxylin/Eosin or processed for immunohistochemistry. To determine metastatic burden, number and size of metastatic foci was quantified in Hämatoxylin/Eosin (H&E)-stained 100μm serial sections of whole lungs with Aperio Image Scope Software. The metastatic index of an animal was calculated as percentage of collective metastatic area in all sections to total lung area. Total lung area was determined by addition of the areas of individual lung lobes at their biggest section. Metastatic incidence was calculated as percentage of animals with metastasis to total animals. Immune cell aggregates in the lung were quantified in the same way as metastases.

### Immunohistochemistry

For immunohistochemistry, heat-mediated antigen unmasking was performed and incubation with the primary antibody was done as indicated in the following: Anti-Ki-67 1:1500, 30min RT, 12202S (Cell signaling); Anti-cleaved caspase 3 (Asp175) 1:200, 3h RT, 39579S (Cell signaling); Anti-CD3 1:2, 30min RT, IS503 (Dako); Anti-CD68 1:100, 30min RT, ab12521 (abcam); Anti-MPO 1:50, over night 4°C, ab139748 (abcam). Biotin-conjugated secondary antibodies (Vectorlabs) were used for primary antibody detection according to the DAB protocol (Vectorlabs). In tumors, Ki-67 positive cells were quantified automatically with Aperio Image Scope software version 12.3.2.5030 (Leica) in the whole section while cleaved caspase 3 positive cells were counted manually in six random 20x-fields. In lung metastases, Ki-67 and cleaved caspase 3 positive cells were counted manually and were depicted as positive cells per mm2 with respect to the area of the corresponding metastases.

CD3-, CD68- and MPO-staining in lung parenchyma were quantified in nine random 40x-fields from three different lung lobes. Positive cells were manually counted in ImageJ 1.49c. Normalization to a reference image with a given amount of lung tissue was performed since amount of lung tissue in a 40x-field could differ.

### Gene expression analysis in tumors, lungs, mammary glands and blood

For mRNA expression analysis tumor, lung, mammary gland or blood tissue were homogenized in RLT lysis buffer (Qiagen) with one 20s pulse at 5000rpm of the precellys 24 homogenizator (bertin instruments). Lysates were centrifuged at 4°C and 13.000 rpm for 5min. RNA was isolated from lysates with RNeasy Mini Kit (Qiagen) according to the manufacturer's protocol. cDNA synthesis was performed with 0,5 to 1μg of RNA and SuperScript II Reverse Transcriptase (Invitrogen) according to the manufacturer's protocol. cDNA level were subsequently determined by quantitative real time PCR with a SYBR-Green MasterMix (Roche) on a StepOnePlus Real Time PCR system (Applied Biosystems). Expression levels were normalized based on the expression of housekeeping gene cyclophilin. The following primers were used:

**Table:** 

Il1b fwd	TGTCTTTCCCGTGGACCTTC
Il1b rev	CCCATGAGTCACAGAGGATGG
Ptgs2 fwd	AGACAGATCATAAGCGAGGACC
Ptgs2 rev	CCATCCTTGAAAAGGCGCAGT
Twist1 fwd	GTCCCACTAGCAGCGGAG
Twist1 rev	TGTCCATTTTCTCCTTCTCTGGAA
Cdh1 fwd	AACGCTCCTGTCTTCAACCC
Cdh1 rev	GGTCACTTTGAGTGTGGCGA
Hif1a fwd	ACACAGAAATGGCCCAGTGA
Hif1a rev	GGCTTGTTAGGGTGCACTTC
Ifng fwd	TTACTGCCACGGCACAGTCA
Ifng rev	AGTTCCTCCAGATATCCAAGAAGAGA
Vim fwd	ACGAGTACCGGAGACAGGTG
Vim rev	AGTTAGCAGCTTCAAGGGCAA
Ctsl fwd	TTCAGGAACCGCTGATGCTT
Ctsl rev	CTGTCCTTCTAGGCAACCCG
Ccl17 fwd	AATGTAGGCCGAGAGTGCTG
Ccl17 rev	GCCCTGGACAGTCAGAAACA
SnaI1 fwd	GTCTGCACGACCTGTGGAAA
SnaI1rev	GGTCAGCAAAAGCACGGTTG
Ctss fwd	GGACTGGAGAGAGAAGGGCT
Ctss rev	AGCTTCCCCGTTTTCAGCTT
Tgfb1 fwd	TGTGGAGCAACATGTGGAACT
Tgfb1 rev	TGCCGTACAACTCCAGTGAC
Snai2 fwd	GCACATTCGAACCCACACATT
Snai2 rev	TGCAGTGAGGGCAAGAGAAAG
Cxcl1 fwd	ACTGCACCCAAACCGAAGTC
Cxcl1 rev	TGGGGACACCTTTTAGCATCTT
Tnfa fwd	CCCACGTCGTAGCAAACCA
Tnfa rev	GTACAACCCATCGGCTGGC
Mmp14 fwd	CCAAGGCAGCAACTTCAGC
Mmp14 rev	GTGAGCGTTGTGTGTGGGTA
Csf1 fwd	GCCAAGGAGGTGTCAGAACA
Csf1 rev	AGGCAATCTGGCATGAAGTCT
Postn fwd	CCCGCAGTGATGCCTATTGA
Postn rev	CAGCTTCGAGACATCGGAGT
Gzmb fwd	CCCAAAGACCAAACGTGCTT
Gzmb rev	CGTGGAGGTGAACCATCCTTA
Cccl5 fwd	TGCCCACGTCAAGGAGTATTT
Cccl5 rev	TTCTCTGGGTTGGCACACA
S1008a fwd	CAAGGAAATCACCATGCCCT
S1008a rev	ATATTCTGCACAAACTGAGGACAC
S1009a fwd	CCTGACACCCTGAGCAAGAA
S1009a rev	GTCCTGGTTTGTGTCCAGGT
Tnc fwd	AGCTTCCTGCTTAAGTCCCTG
Tnc rev	TAGGTGGCACACAACTGCTC
Fn1 fwd	AAGAGGCAGGCTCAGCAAAT
Fn1 rev	GTCCGTTCCCACTGCTGATT
Csf2 fwd	TCAAAGAAGCCCTGAACCTCC
Csf2 rev	CGAATATCTTCAGGCGGGTCT
Ccl2 fwd	CAGCCAGATGCAGTTAACGC
Ccl2 rev	AGCCTACTCATTGGGATCATCTTG
Ccl22 fwd	TCATGGCTACCCTGCGTGTC
Ccl22 rev	CCTTCACTAAACGTGATGGCAGAG
Vegfa fwd	ACCCACGACAGAAGGAGAGC
Vegfa rev	GGTCTCAATCGGACGGCA
Mmp1 fwd	ACGCTGCTTATGAAGCTAGTATGA
Mmp1 rev	TCTCTGGGGAATCCTCTCAGT
Cxcl12 fwd	CGGTTCTTCGAGAGCCACAT
Cxcl12 rev	TTGTTGTTCTTCAGCCGTGC

### Flow cytometric analysis of immune cell populations

To characterize myeloid cell populations in tumors, tumor tissue was isolated and minced with a scalpel. Tissue pieces were enzymatically digested for 40min at 37°C and moderate shaking with 2mg/ml Collagenase (Sigma-Aldrich) and 100μg/ml DNase I (Roche) in 10ml DMEM with 2% fetal calve serum (Gibco). After digestion, 40ml PBS (Gibco) was added and the suspension was filtered through 0.7μm nylon strainers (Thermo Fisher Scientific). The suspension was centrifuged for 5min at 4°C and 500x g and the supernatant was discarded. The cell pellet was resuspended in red blood cell lysis buffer (Sigma-Aldrich) and incubated for 10min at room temperature. 40ml of PBS were added and cells were centrifuged again. Cells were resuspended in staining buffer containing 1:1000 efluor780 viability dye (Thermo Fisher Scientific), 1:200 CD16/CD32 Fc-blocking antibody (eBioscience) and 1:200 fluorochrome-conjugated antibodies.

**Table:** 

Antigen	Antibody	Fluorochrome
CD45	eBioscience, 30-F11	PerCP-Cyan5.5, efluor450
CD11b	BD bioscience, M1/70	BV500, PE-Cy7
Gr1	eBioscience, RB6-8C5	APC
F4/80	eBioscience, BM8	PE
CD3	eBioscience, 17A2	APC, BV605
CD8b	BD bioscience, H35-17.2	PE, APC
CD4	eBioscience, RM4-5	efluor450, BV510
B220	eBioscience, RA3-6B2	FITC
Ifn-y	eBioscience, XMG1.1	PerCP Cy-5.5.
GrzmB	eBioscience, NGZB	FITC
NKp46	BD bioscience, 29A1.4	A700

After 20min of incubation with the primary antibody at room temperature in the dark, cells were washed twice with PBS and fixed in IC fixation buffer (eBioscience). Cells were washed again in PBS and analyzed by FACS. Staining of lymphoid cell populations in tumor and lung tissue was performed in the same way, however, mononuclear cells were purified prior to staining with density centrifugation in a three-layered Percoll (GE Healthcare) gradient (30%, 40% and 75%). The cell solution was carefully applied on top of the gradient and centrifuged for 20min at 220x g at 4°C with disabled rotor break. The interphase containing the mononuclear cells was isolated, washed with PBS and pelleted. For cytokine staining, purified mononuclear cells were stimulated for 4h at 37°C and 5%CO_2_ in RPMI (Gibco) with 10% fetal calf serum (Gibco), PMA (20ng/ml) (Sigma-Aldrich), Ionomycin (1ug/ml) (Sigma-Aldrich) and GolgiPlug (1:1000) (BD bioscience). Cells were pelleted and resuspended in staining buffer with antibodies against surface antigens for 20min at RT, washed with PBS (Gibco) containing 2% fetal calf serum (Gibco) and fixed in IC fixation buffer (eBioscience). Cells were washed with 1x permeabilization buffer (eBioscience) and intracellular staining was performed over night at 4°C. Cells were then washed again and analyzed by FACS.

### Relative quantification of circulating tumor cells

Blood from tumor-bearing animals was collected by cardiac puncture and transferred to an EDTA-containing S-monovette (Sarstedt) to prevent blood clotting. 5ml red blood cell lysis buffer was added to up to 500μl of blood. After 10min at room temperature, lysis buffer was diluted with 45ml of PBS and the cells were pelleted by centrifugation at 4°C and 500x g for 5min. The supernatant was aspirated and the cell pellet was resuspended in RLT lysis buffer (Qiagen) including 1% β-mercaptoethanol (Sigma-Aldrich). Samples were then stored at −80°C until RNA isolation. RNA isolation was performed as described above. On-column DNA digestion during isolation as well as additional off-column DNAse I digest was performed with 1U/μl RNAse-free DNAse I (Thermo Fischer Scientifc) for 15min at 37°C to remove residual genomic DNA. Digestion was stopped by heat inactivation at 65°C for 10min.

### Peripheral blood count

Blood was collected from the facial vein and analyzed with a scil vet abc blood counter (scil).

### Co-culture of tumor cells with lung tissue

PyMT tumor-derived TS1 tumor cells [61] were seeded on a 24 well plate at a density of 15.000 or 50.000 cells per well. On the next day, PyMT *Ikkβ^F/F^* or PyMT *Ikkβ^Δmye^* animals were sacrificed and their lungs were perfused with PBS via the right ventricle to flush out the blood. Lungs were isolated, lobes were separated and tissue was cut in equal pieces. Tumor cells were washed once with PBS and serum-reduced DMEM medium with Pen/Strep (Gibco), Glutamax (Gibco) and 0,1% fetal calve serum (Gibco) was added to each well. Transwells with a pore size of 0,4μM (Greiner Bio-One) were placed in the well and 40mg of lung tissue was placed in the upper compartment of the transwells in reduced DMEM. Cells and lung tissue were co-cultured over night for 15h, then, the lung tissue pieces were removed and cells were incubated for another 24h. Afterwards, cells were stained with trypan blue (Sigma-Aldrich) and counted with a hemocytometer (Laboroptik). To compare different experiments, the number of cells after co-culture with lung tissue from PyMT *Ikkβ^F/F^* or PyMT *Ikkβ^Δmye^* mice was normalized to the number of control cells seeded and grown in parallel at the corresponding density and time in complete DMEM with Pen/Strep (Gibco), Glutamax (Gibco) and 10% fetal calve serum (Gibco). For each of the three experiment depicted, four to five replicates per condition existed.

To determine cell cycle stage and viability of TS1 cells after co-culture with lung tissue, co-culture was performed as described above but the experiment was scaled up to yield an appropriate amount of cells for FACS analysis. 70.000 TS1 cells per well were plated in a 6 well plate and co-cultured with ≈90mg of lung tissue per well in 3,5ml serum-reduced DMEM medium. For each condition two replicates existed. After the incubation period, cells were trypsinized and their concentration was adjusted to a number of 0,5×106 cells/ml in DMEM with 2% FCS, 1:10 Penicilin/Streptomycin, Glutamax, 10mM Hepes and 5ug/ml Hoechst 33342 dye (Sigma-Aldrich). Cells were incubated for 30 min in a 2ml reaction tube in the water bath at 37°C and were mixed by inverting every 10min. Cells were then centrifuged for 5min at 500g and 4°C and resuspended in ice-cold Hanks buffered salt solution with 10mM HEPES and 2% FCS (HBSS+) with 2ug/ml propidium iodide (ebioscience). Cells were incubated on ice for 20min, washed once with HBSS+ buffered salt solution with 10mM HEPES and 2% FCS and analyzed by FACS.

### Statistics

Statistics of data depicted in the figures was calculated with PRISM7 (Graphpad). When comparing two data sets, unpaired, two-tailed *t*-test was performed. When comparing more than two data sets, one-way ANOVA and Bonferroni's multiple comparison were performed. For metastatic incidence no statistics was done since a single value is depicted in the graph (percentage). Differences in datasets are considered significant only when a p value smaller than p = 0,05 is indicated as in the following: ^*^p < 0,05, ^**^p < 0,01, ^***^p < 0,001, ^****^p < 0,0001.

## SUPPLEMENTARY MATERIALS FIGURES



## Abbreviations

BMDMs: bone marrow-derived macrophages
EMT: epithelial to mesenchymal transition
GRA: granulocytes
LYM: lymphocytes
MON: monocytes
PI: propidium iodide
PyMT: MMTV polyoma middle T
TAMs: tumor-associated macrophages
TANs: Tumor-associated neutrophils
WBC: white blood cells
