# Supplementary material for: Canonical NF-κB signaling in myeloid cells promotes lung metastasis in a mouse breast cancer model
Source: Oncotarget. 2018 Mar 30;9(24):16775–91. doi: 10.18632/oncotarget.24697 (PMC5908285; doi:10.18632/oncotarget.24697)
Supplement: Supplementary file 1 [file oncotarget-09-16775-s001.pdf]

Canonical NF-κB signaling in myeloid cells promotes lung metastasis in a mouse breast cancer model

SUPPLEMENTARY MATERIALS

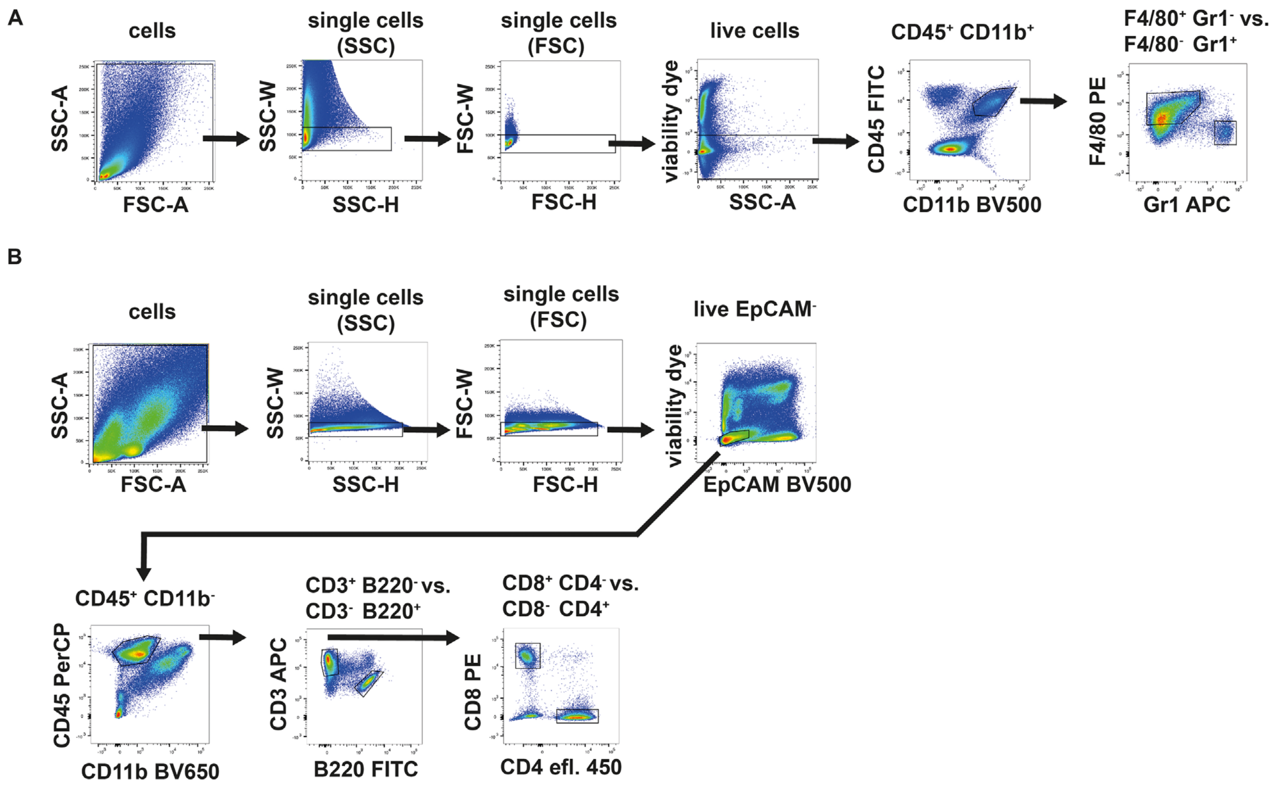

Supplementary Figure 1: FACS gating strategy of tumor infiltrating cells. Gating strategy of **(A)** myeloid and **(B)** lymphoid immune cells in tumors of PyMT *Ikkβ*$^{F/F}$ and PyMT *Ikkβ*$^{\Delta mye}$ mice displayed in Figure 2A. SSC=side scatter, FSC=forward scatter, A=area, H=height, W=width.

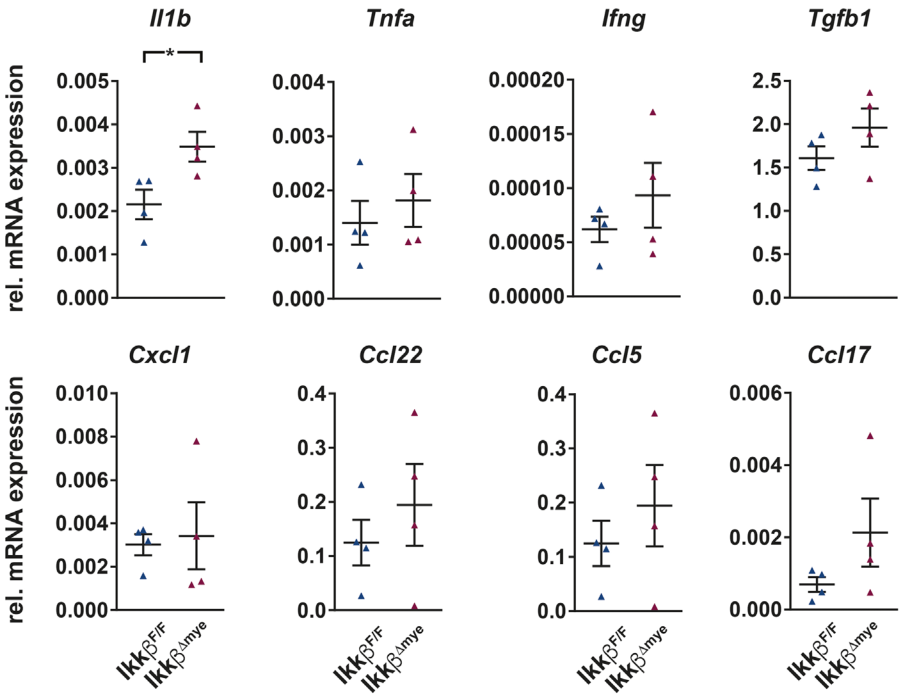

Supplementary Figure 2: Expression of inflammatory mediators in the mammary gland. Quantitative PCR of the indicated genes from mammary gland tissue of tumor free *Ikkβ*$^{F/F}$ and *Ikkβ*$^{\Delta mye}$ mice (n=4) at 8 weeks of age. Tissue from three mammary glands per animal was analyzed. Data are mean ± SEM $^{*}p \leq 0,05$.

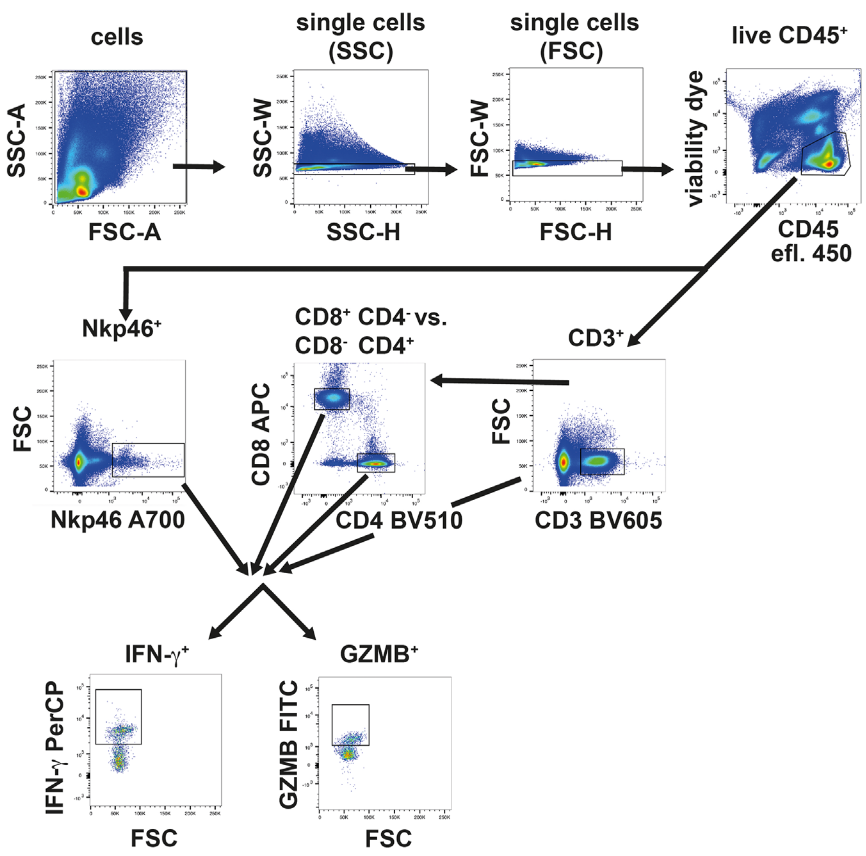

Supplementary Figure 3: FACS gating strategy of IFNy and GZMB expressing cells in the lung. Gating strategy of IFN-γ and Granzyme B expressing mononuclear cells from lungs of PyMT $Ikk\beta^{F/F}$ and PyMT $Ikk\beta^{\Delta mye}$ mice displayed in Figure 5. SSC=side scatter, FSC=forward scatter, A=area, H=height, W=width.
